# Genomic Characterization of Novel Human Parechovirus Type

**DOI:** 10.3201/eid1502.080341

**Published:** 2009-02

**Authors:** Linlin Li, Joseph Victoria, Amit Kapoor, Asif Naeem, Shahzad Shaukat, Salmaan Sharif, Muhammad Masroor Alam, Mehar Angez, Sohail Zahoor Zaidi, Eric Delwart

**Affiliations:** Blood Systems Research Institute, San Francisco, California, USA (L. Li, J. Victoria, A. Kapoor, E. Delwart); University of California, San Francisco (L. Li, J. Victoria, A. Kapoor, E. Delwart); National Institute of Health, Islamabad, Pakistan (A. Naeem, S. Shaukat, S. Sharif, M.M. Alam, M. Angez, S.Z. Zaidi)

**Keywords:** Pakistan, parechovirus, genomic, human, HPeV, dispatch

## Abstract

Using a simple metagenomic approach, we identified a divergent human parechovirus (HPeV) in the stool of a child in Pakistan. Genomic characterization showed this virus was distinct enough from reported HPeV types to qualify as candidate prototype for the seventh HPeV type.

Human parechoviruses (HPeVs) belong to the recently identified genus *Parechovirus* of the family *Picornaviridae*. Serologic and molecular studies show that HPeV comprises 6 neutralization serotypes or corresponding types based on capsid protein similarities, HPeV1–6. HPeV1 and HPeV2, originally known as enterovirus echoviruses 22 and 23, were isolated in 1956 (*1*). Because echoviruses 22 and 23 had serologic, molecular, and biologic properties highly distinct from other enteroviruses, they were reclassified in 1999 as members of the genus *Parechovirus* (*2*). The other 4 parechovirus types were identified more recently from young children with clinical manifestations similar to those caused by human enteroviruses: HPeV3 in 2002 (*3*), HPeV4 in 2005 (*4*), and HPeV5 (*5*) and HPeV6 (*6*) in 2006. HPeV infections occur commonly in the general population and mostly cause mild gastrointestinal and respiratory symptoms in young children (*7*). More severe consequences also have been ascribed to HPeV infections, including acute flaccid paralysis (AFP) (*3*), encephalitis (*8*), aseptic meningitis (*9*), myocarditis (*10*), neonatal sepsis (*11*), and Reye syndrome (*6*),

Nonpolio AFP may be caused by many viruses, including nonpolio enteroviruses, human adenoviruses, herpes simplex, Epstein-Barr virus, and West Nile virus (*12*). HPeV1 was associated with an AFP outbreak in Jamaica in 1986. In 2 of 3 AFP patients with HPeV1 detected in stool samples, antibody titer also increased significantly (*13*). HPeV6 was isolated from the stool specimen of an AFP patient in Japan in 2001 (*6*). HPeV3 has not been reported from AFP cases but was identified in 1 transient paralysis case and believed to cause serious central nervous system symptoms more frequently than HPeV1 (*3**,**7*). HPeV types 2, 4, and 5 have been less often observed in clinical studies.

Using sequence-independent PCR amplification and sequence similarity searches, we recently investigated virus sequences in stool samples from children in Pakistan who had nonpolio AFP and from healthy children who had close contacts with persons who had AFP. Sequences of human parechoviruses were identified in samples from 6 of 65 persons. Analysis showed 5 HPeV infections in 56 samples from persons who had nonpolio AFP, 1 HPeV1, 1 HPeV5, and 2 HPeV6; in 1 sample, HPeV type could not be determined because the sequenced fragment was located in a phylogenetically uninformative region. A highly divergent HPeV type also was identified in 1 contact sample, and the full genome of this virus was sequenced. Phylogenetic analysis indicated that this virus, designated PAK5045, has the genetic characteristics expected of a new HPeV type.

## The Study

HPeV PAK5045 was found in 1 stool sample from a healthy 2-year-old boy who had close contact with a person who had nonpolio AFP, using a previously described method applied here to stool samples (*14*). Briefly, virus nucleic acids were purified from stool samples, randomly amplified by reverse transcription (RT)–PCR using 3′ randomized RT and PCR primers, subcloned, and sequenced. HPeV sequences were abundant in 1 sample with 24/48 plasmid subclones identified as PAK5045. Assembly of these HPeV sequences produced 5 fragments covering ≈75% of the genome. Specific PCR primers were used to link these genome fragments, and rapid amplification of cDNA ends was carried out to acquire the 5′ and 3′ ends.

The nucleotide sequence of PAK5045 virus was 7,127 nt, excluding a poly (A) tail. PAK5045 contained a partial 5′ untranslated region (UTR) of 511 nt, an open reading frame (ORF) encoding a putative polyprotein precursor of 2,175 aa, and a 3′ UTR of 88 nt. The nucleotide sequence of PAK5045 was generated with at least 2× coverage except for the 5′ UTR. The full-length sequence of PAK5045 has been deposited in GenBank under accession no. EU556224.

The polyprotein of PAK5045 comprised capsid proteins VP0 (289 aa), VP3 (254 aa), and VP1 (226 aa) and nonstructural proteins 2A (149 aa), 2B (122 aa), 2C (329 aa), 3A (117 aa), 3B (20 aa), 3C (200 aa), and 3D (469 aa). Comparison of the complete ORF of PAK5045 with the 6 HPeV prototypes showed it was closely related to HPeVs and had amino acid identity of 84.8%–89.1% and nucleotide identity of 75.6%–80.8% (Table 1). Of the 6 known HPeV types, the intertype amino acid identities ranged from 84.9% to 91.1%, and the intertype nucleotide identities of the ORF sequence ranged from 76.1% to 83.4% (data not shown), a range similar to their identities relative to PAK5045.

**Table 1:** Nucleotide and amino acid sequence comparisons of HPeV7 candidate prototype PAK5045 with the HPeV prototypes*

Sequence	% Nucleotide (amino acid) identity					
	HPeV1	HPeV2	HPeV3	HPeV4	HPeV5	HPeV6
5′ UTR	87.6	86.8	95.9	90.2	86.7	89.8
VP0	71.0 (76.8)	69.6 (75.8)	68.3 (72.3)	69.7 (75.1)	70.1 (75.4)	69.7 (73.7)
VP3	69.5 (75.3)	69.9 (74.1)	73.2 (83.9)	70.3 (77.5)	69.7 (74.0)	68.8 (74.0)
VP1	65.8 (69.9)	66.6 (69.5)	68.9 (77.0)	67.8 (68.6)	64.7 (66.4)	63.9 (65.0)
2A	77.9 (89.3)	75.2 (84.6)	77.5 (85.9)	80.8 (89.3)	80.5 (89.9)	76.5 (85.9)
2B	80.1 (95.9)	76.8 (95.1)	86.1 (99.2)	83.6 (99.2)	83.6 (97.5)	78.7 (96.7)
2C	78.9 (91.5)	76.5 (86.6)	83.8 (96.0)	85.9 (97.0)	80.9 (94.5)	79.5 (90.0)
3A	76.6 (88.0)	76.1 (83.8)	81.8 (94.0)	87.5 (96.6)	78.9 (82.9)	76.1 (89.7)
3B	73.3 (90.0)	75.0 (90.0)	76.7 (85.0)	85.0 (95.0)	76.7 (90.0)	70.0 (90.0)
3C	81.3 (98.0)	81.2 (98.0)	83.5 (98.0)	86.3 (98.0)	80.3 (99.0)	83.0 (99.0)
3D	83.2 (94.9)	82.8 (94.0)	88.7 (96.8)	90.8 (97.2)	83.8 (95.9)	83.6 (95.3)
3′ UTR	85.1	89.8	95.5	94.4	85.2	83.0
ORF	76.3 (86.7)	75.6 (84.8)	79.5 (89.1)	80.8 (88.5)	77.0 (86.6)	76.0 (85.4)

*Sequences for the HPeV prototypes, HPeV1 (Harris, S45208), HPeV2 (Williamson, AJ005695), HPeV3 (A308/99, AB084913), HPeV4 (K251176-02, DQ315670), HPeV5 (CT86-6760, AF055846), and HPeV6 (NII561–2000, AB252582), were obtained from GenBank. HPeV, human parechoviruses.

**Figure 1:**
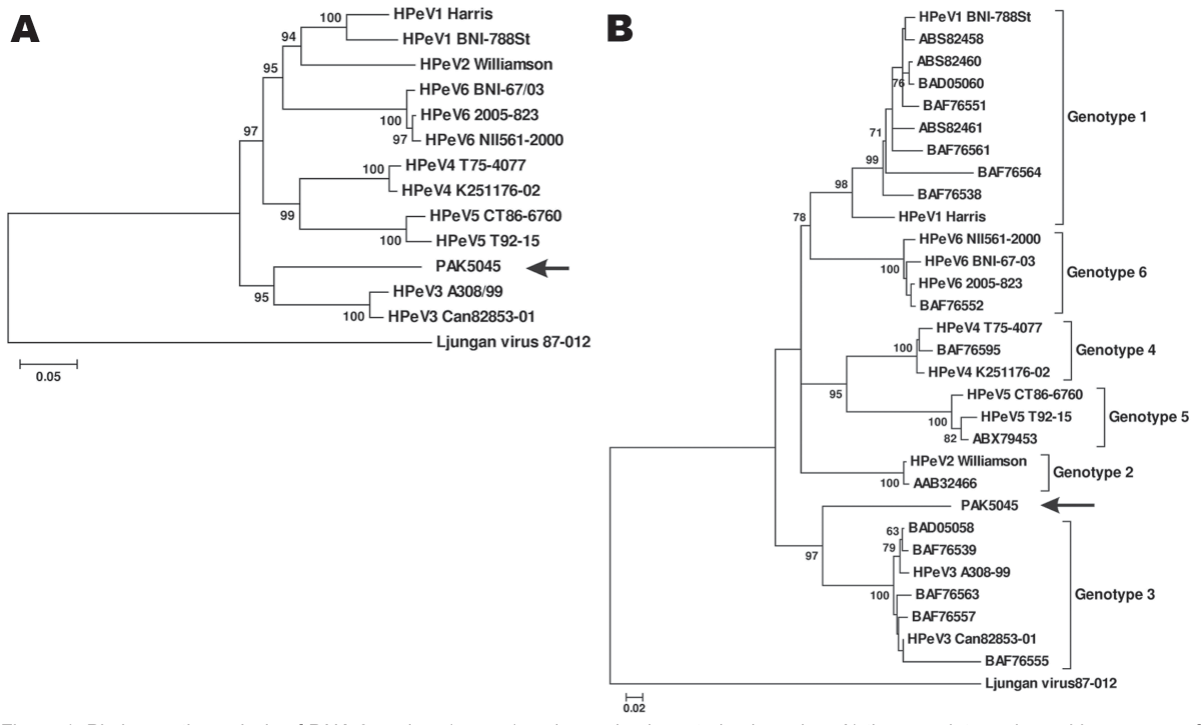
Phylogenetic analysis of PAK5045 virus (arrows) and parechovirus strains based on A) the complete amino acid sequence of P1 region and B) the complete amino acid sequence of viral protein 1 (VP1). All parechovirus sequences were obtained from GenBank, including 13 completely sequenced parechoviruses, human parechoviruses (HPeV) 1 Harris (Q66578), HPeV1 BNI-788St (ABK54353), HPeV2 Williamson(CAA06679), HPeV3 A308/99(BAC23086), HPeV3 Can82853-01(CAI64373),HPeV4 K251176-02 (ABC41566), HPeV4 T75-4077 (CAJ84484), HPeV5 CT86-6760 (Q9YID8), HPeV5 T92-15 (CAJ84483),HPeV6 NII561-2000 (BAF63403), HPeV6 2005-823 (ABX79460), HPeV6 BNI-67/03 (ABS82455), Ljungan virus 87-012 (AAM46079), and 17 sequences with accession numbers shown directly on the tree. Both trees were constructed by the neighbor-joining method with 1,000 bootstrap replicates using MEGA4.

Phylogenetic analysis with the complete P1 amino acid sequences of fully sequenced HPeVs confirmed the existence of the 6 types defined by previous studies (Figure 1, panel A) (*15*). PAK5045 virus was most similar to HPeV3 strains. The identity of P1 amino acid sequences between PAK5045 virus and both HPeV3 strains analyzed was 77.5%, which was lower than some HPeVs intertype amino acid identities (e.g., average of 82.4% between HPeV1 and HPeV2, 78.3% between HPeV1 and HPeV4, 80.4% between HPeV1 and HPeV6, 78.4% between HPeV2 and HPeV6, and 80.5% between HPeV4 and HPeV5) (data not shown).

Consistent with the P1 amino acid tree, phylogenetic analysis with VP1 capsid proteins also showed 6 established types (Figure 1, panel B) (*5*). PAK5045 VP1 was slightly closer to type 3 strains, with the greatest amino acid identity being 78.8% (Table 2), and more divergent from the other established HPeV types. We retrieved from GenBank, and then analyzed, genetic relationships among 92 full-length VP1 amino acid sequences and with PAK5045. None clustered with PAK5045 as a close genetic lineage. The amino acid identities between PAK5045 and HPeV3 strains ranged from 69.9% to 78.8%, outside the HPeV3 intratype range of 85.8%–100% (Table 2).

**Table 2:** VP1 amino acid sequence comparisons of HPeV types*

Type	Average (range) of VP1 amino acid identities, %						
	HPeV1	HPeV2	HPeV3	HPeV4	HPeV5	HPeV6	PAK5045
HPeV1	94.7 (81.8–100.0)	78.3 (71.7–80.0)	71.2 (61.9–73.9)	75.6 (69.7–78.4)	72.6 (66.7–74.9)	78.2 (71.9–81.8)	68.1 (64.6–69.9)
HPeV2		99.4 (99.1–100.0)	71.4 (65.5–72.6)	74.9 (74.3–76.1)	71.8 (70.4–72.6)	73.7 (73.5–73.9)	69.5 (–)
HPeV3			97.3 (85.8–100.0)	70.8 (66.4–71.7)	66.8 (60.6–68.6)	73.9 (66.8–75.7)	77.6 (69.9–78.8)
HPeV4				97.0 (96.6–97.8)	78.3 (75.9–80.2)	72.8 (72.3–73.2)	68.4 (68.1–68.6)
HPeV5					96.9 (94.8–100.0)	72.0 (71.0–72.7)	66.1 (65.5–66.4)
HPeV6						97.2 (95.7–98.7)	64.9 (64.6–65.0)
PAK5045							–

*92 full-length VP1 amino acid sequences were obtained from GenBank, including 46 HPeV1, 4 HPeV2, 31 HPeV3, 3 HPeV4, 4 HPeV5 and 4 HPeV 6 VP1 amino acid sequences. The accession nos. are AAA72291, AAB32466, AAB23363, AAC79756, ABC41566, ABK54353, ABS82455, ABS82457–82462, ABX79460, ABX79453, BAC23086, BAD05057–05062, BAF63403, BAF76536–6596, CAA06679, CAI64373, CAJ84483~84484, NP_046804, NP_740386, Q66578, and Q9YID8. HPeV, human parechoviruses; VP1, viral protein 1.

The PAK5045 polyprotein contained 9 putative cleavage sites at VP0/VP3 (T/A), VP3/VP1 (Q/N), VP1/2A (E/S), 2A/2B (Q/G), 2B/2C (Q/G), 2C/3A (Q/T), 3A/3B (E/R), 3B/3C(Q/R), and 3C/3D (Q/G). Alignments showed that VP3/VP1, VP1/2A, and 2C/3A cleavage sites differed for PAK5045 relative to those of fully sequenced HPeVs strains, whereas the other 6 sites were conserved. The cleavage site in VP0/VP3 of PAK5045 was identical to that of HPeV2 but not to those of other types. The VP1/2A cleavage site was identical between PAK5045 and HPeV3 strains A308/99 and Can82853-01 but not other HPeVs. The RGD motif (arginine-glycine-aspartic acid) at the C terminus of VP1 was absent in PAK5045 and in HPeV3 strains A308/99 and Can82853-01, which indicates that mechanisms other than RGD binding to integrins may occur during PAK5045 infection.

**Figure 2:**
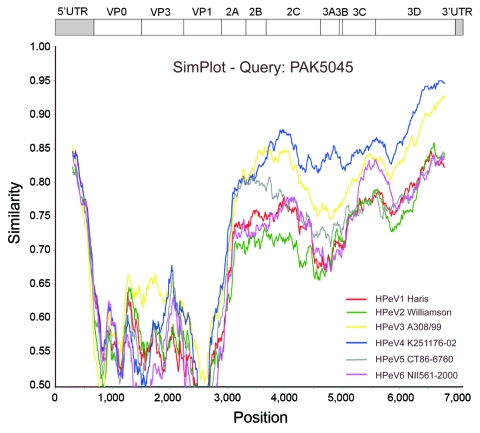
SimPlot (http://sray.med.som.jhmi.edu/SCRoftware/simplot) analysis of the full-length sequences of the human parechovirus (HPeV) prototypes against PAK5045 query, based on nucleotide similarities. Each curve compares the PAK5045 genome with an HPeV prototype. The Kimura 2-parameter model was applied with a transition/transversion (Ts/Tv) ratio of 3.0 (*5*), and a sliding window of 600 nt with a step size of 10 nt was used.

To identify recombination events between the different HPeV types, we performed SimPlot analysis (http://sray.med.som.jhmi.edu/SCRoftware/simplot) of the 6 complete nucleotide HPeV prototype genomes against PAK5045 (Figure 2). In general, PAK5045 was closer to HPeV3 and HPeV4 than to the other viruses. PAK5045 showed a relatively higher degree of nucleotide similarity to HPeV3 A308/99 in the P1 region consistent with the P1 phylogenetic tree. Downstream of nucleotide position 3600, HPeV4 K251176-02 became the closest relative of PAK5045 in most of the nonstructural (P2/3) region, which suggests an ancient recombination event.

## Conclusions

We identified and characterized a novel HPeV type from the stool sample of a healthy children who had been in close contact with person who had nonpolio AFP. The genome sequence diverged sufficiently from the 6 known HPeVs to qualify as a candidate for the prototype of HPeV7. Using only a low-level shotgun sequencing method, we detected HPeVs in 9% (6/65) of stool samples from patients with nonpolio AFP, including HPeV types 1, 5, 6, and 7. A more sensitive method, such as HPeV-directed RT-nested PCR, is likely to have detected a higher prevalence. The median age of sampled patients was 3 years (range 1 month–15 years), and all HPeV-positive patients were <3 years of age.

In a previous study, HPeVs were isolated from 0.3% of 13,656 various clinical samples collected in Japan (14 HPeV1, 16 HPeV3, 10 HPeV6, and 1 HPeV4) (*6*). In Germany, the detection rate of HPeVs did not differ significantly between patients with acute diarrhea and controls, with 11.6% (7/60) of children <2 years of age being HPeV positive (*15*). In a Dutch study of 303 isolates showing cytopathic effects consistent with enterovirus infection, 12% were HPeV positive, with 27 HPeV1 and 10 HPeV3, all in children <3 years of age (*7*). HPeV infection, therefore, seems to be associated with young children (<3 years). More studies are needed to associate HPeV infection (with any genotypes) with development of neurologic disease, such as AFP.
